# Exploring the Influence of Solvents on Electrochemically Etched Porous Silicon Based on Photoluminescence and Surface Morphology Analysis

**DOI:** 10.3390/ma17050989

**Published:** 2024-02-21

**Authors:** Meng-Ting Tsai, Yi-Chen Lee, Yung-Mei Lin, Vincent K. S. Hsiao, Chih-Chien Chu

**Affiliations:** 1 Department of Medical Applied Chemistry, Chung Shan Medical University, Taichung 40201, Taiwan; s0564036@gm.csmu.edu.tw; 2 Department of Applied Materials and Optoelectronic Engineering, National Chi Nan University, Nantou 54561, Taiwan; s110328011@ncnu.edu.tw (Y.-C.L.); s110328053@ncnu.edu.tw (Y.-M.L.); 3 Department of Medical Education, Chung Shan Medical University Hospital, Taichung 40201, Taiwan

**Keywords:** porous silicon, photoluminescence, electrochemical etching, solvent effects, surface morphology, emission characteristics, optoelectronic devices

## Abstract

Porous silicon (PSi) has promising applications in optoelectronic devices due to its efficient photoluminescence (PL). This study systematically investigates the effects of various organic solvents and their concentrations during electrochemical etching on the resulting PL and surface morphology of PSi. Ethanol, n-butanol, ethylene glycol (EG) and N,N-dimethylformamide (DMF) were employed as solvents in hydrofluoric acid (HF)-based silicon etching. The PL peak position exhibited progressive blue-shifting with increasing ethanol and EG concentrations, accompanied by reductions in the secondary peak intensity and emission linewidth. Comparatively, changes in n-butanol concentration only slightly impacted the main PL peak position. Additionally, distinct morphological transitions were observed for different solvents, with ethanol and n-butanol facilitating uniform single-layer porous structures at higher concentrations in contrast to the excessive etching caused by EG and DMF resulting in PL quenching. These results highlight the complex interdependencies between solvent parameters such as polarity, volatility and viscosity in modulating PSi properties through their influence on surface wetting, diffusion and etching kinetics. The findings provide meaningful guidelines for selecting suitable solvent conditions to tune PSi characteristics for optimized device performance.

## 1. Introduction

While investigating the polishing of silicon wafers with hydrofluoric acid (HF) as the electrolyte at Bell Labs in 1956, a notable discovery occurred when Uhlir accidentally observed the formation of a black film covering the wafer’s surface [1]. This intriguing phenomenon was later ascribed to the creation of a porous structure resulting from the dissolution of silicon, subsequently named porous silicon (PSi). Experimental results revealed that diverse etching patterns could be achieved by varying the current densities while maintaining a constant HF concentration. The revelation of room-temperature photoluminescence (PL) in PSi under optical excitation dates back to Pickering’s report in 1984 [2]. The observed excitation wavelength and intensity were found to be influenced by experimental parameters, such as the etching solution concentration, current density and etching time. Nonetheless, during this period, no theoretical framework was proposed to explain this phenomenon. In 1990, Canham introduced the quantum-confinement model to elucidate the PL observed in PSi [3]. According to Canham’s findings, the above-bandgap luminescence observed in freshly anodized Si substrates is attributed to a quantum size effect rather than an alloying effect in an amorphous phase. Notably, PL is not observed in macroporous layers in n-substrates with Si skeleton dimensions too large to expect quantum size effects. Also, the significant increase in PL intensity during simple chemical dissolution in aqueous HF further supports the quantum size effect hypothesis. This phenomenon suggests that the luminescence arises from the PSi skeleton rather than an amorphous phase. Since Canham’s initial proposal, our understanding of the PL from PSi has evolved significantly. While the quantum-confinement model remains a cornerstone, ongoing research has expanded our knowledge by investigating the influence of various factors such as surface passivation, surface recombination velocities and fabrication techniques on the tuning of the PL properties. These findings paved the way for diverse applications utilizing PSi as a substrate, spanning technologies such as light-emitting diodes [4,5], photodetectors [6,7,8,9,10,11], solar cells [12,13,14,15,16] and biomedical applications [17,18,19,20].

The formation of pores in PSi is believed to come from the irregular distribution of the electric current at the interface between the silicon and HF [2]. This irregularity induces the creation of small pits where the local current density is higher. The heightened electric field enhances the rate of silicon dissolution, ultimately leading to the development of pores on the surface [21]. Resistance measurements of PS reveal values that are significantly larger than those of the silicon substrate and the solution, indicating that the current tends to enter the pores and flow through the silicon substrate via the pore tips, facilitating silicon dissolution [22]. Consequently, PSi is selectively formed in regions traversed by the electric current. This mechanism involves a complex interplay of factors, including the local current density, the electric field strength and the concentration of the HF solution, contributing to the diverse structures observed in PSi, such as irregular pores, sponge-like formations and dendritic structures [21].

The essential crystalline nature of PSi is observed locally, where the surface silicon oxide constituents do not contribute to photoluminescence (PL) [23]. Notably, the luminescence observed in PSi differs in origin from that found in pure siloxene, HF-treated siloxene, or thermally annealed siloxane [24]. The PL in PSi is attributed to indirect radiative recombinations of excitons localized within the undulating quantum dots formed by crystalline silicon. Additionally, the PL is associated with internal transitions of certain chemical compounds formed on the pore walls [25]. These compounds may include polysilane chains, SiH_x_ molecules, or siloxane [26]. Moreover, the PL is linked to the radiative recombination of electron–hole pairs trapped within surface states, which result from the disordered surface of crystalline silicon in PSi [27].

PSi has gained widespread use in various optoelectronic devices due to its exceptional luminescent properties, large surface-to-volume ratio, and the ability to tune the pore size and distribution [28]. Electrochemical anodization stands out as a simple and rapid method for effectively producing PSi structures. Initially, silicon wafers were etched using an HF solution; however, this resulted in uneven PSi with an unstable current and the generation of a considerable amount of hydrogen gas through hydrolysis, posing safety concerns in large-scale industrial processes [29]. To address these issues, ethanol was introduced to the HF solution, reducing the hydrogen gas produced during hydrolysis [30]. This modification led to smaller bubbles, ensuring a more uniform distribution of the current on the silicon wafer and a more stable etching process. In 2012, Kim investigated an alternative approach by replacing ethanol with isopropyl alcohol (IPA) [31]. IPA not only possesses lower volatility at room temperature but also demonstrates a higher viscosity and lower surface tension. The researchers observed that when the volume ratio of IPA in the etching solution surpasses 50%, the contact angle between the solution and the silicon wafer becomes minimal, facilitating the formation of a uniformly etched surface.

In this work, we conduct a systematic investigation on the effects of various organic solvents, including ethanol, n-butanol, ethylene glycol (EG) and N,N-dimethylformamide (DMF), on the PL and surface morphology of PSi fabricated by HF-based electrochemical etching. The selection of HF–solvent–deionized (DI) water concentrations ranging from 1:1:1 to 1:4:1 was motivated by the aim to investigate a broad range of solvent compositions and their effects on the electrochemical etching of porous silicon PSi. By varying the solvent concentration while keeping the HF and DI water concentrations constant, we aimed to systematically explore the influence of different solvents on the etching process and resultant PSi properties. This allowed for a comprehensive assessment of how changes in solvent composition affect the pore morphology, surface characteristics and PL behavior. The use of varying solvent concentrations enabled us to modulate the etching rate and control the formation of PSi morphology. By comparing the effects of different solvents at equivalent concentration ratios, we aimed to elucidate solvent-specific effects on the etching process. This comparative analysis helped discern unique solvent contributions to PSi formation and provided insights into solvent–surface interactions, dissolution kinetics and surface-passivation effects. Ethanol and n-butanol were found to facilitate uniform etching at higher concentrations, while EG and DMF lead to over-etching. Higher ethanol concentrations effectively mitigate capillary stress-induced fractures, leading to a more homogeneous and controlled nanoscale morphology. The blue shift in the main emission peak, especially under lower concentrations of n-butanol, suggests alterations in the PSi structure or quantum-confinement effects. The presence of secondary emission peaks at specific concentrations further emphasizes the complex interplay between the solvent composition and the resulting optical properties of PSi. The SEM analysis underscores the profound impact of the n-butanol concentration on the surface morphology and defect reduction of PSi. The observed needle-like structures and reduced thickness with increasing n-butanol concentration present opportunities for tailoring the structural characteristics of PSi for diverse optoelectronic applications. The unique properties of n-butanol, particularly its higher viscosity and lower volatility, position it as a promising solvent for achieving controlled and uniform etching of PSi. The solvents induce a progressive blue-shifting of emission peaks and changes in defect-related secondary peaks, as well as a morphological evolution from bi-layer to uniform nanostructured PSi. Further exploration with EG possessing high viscosity and a dielectric constant as a solvent added into the HF etching solution reveals shifting PL signatures from red to eventually no emission at the highest concentration, accompanied by a structural evolution from bimodal pores to solely nanoscale porosity. Investigations on the highly polar solvent DMF highlight the feasibility of obtaining uniform needle-like PSi nanostructures at optimal concentrations, yet over-etching issues arise at higher proportions requiring delicate control, elucidating the complex impacts of solvent polarity and interactions in electrochemical etching. This study not only contributes to the fundamental understanding of the relationship between etching parameters and PL characteristics but also lays the groundwork for enhancing the versatility of PSi in various optoelectronic applications. Future research endeavors may delve into elucidating the underlying mechanisms governing these observed phenomena and further optimizing the conditions for specific device requirements.

## 2. Materials and Methods

The starting material consisted of 500 μm thick, boron-doped, p-type (100) Si wafers (1–10 Ωcm resistivity). Samples measuring 2 × 2 cm^2^ were cleaved using a diamond scribe, then rinsed with DI water and dried with nitrogen gas. The samples were mounted vertically in a Teflon electrochemical cell with the silicon wafer acting as the working electrode (cathode) [32]. A platinum wire counter-electrode (anode) was positioned parallel to the wafer surface and a copper plate contact was made to the back of each sample. The etching solution was prepared with HF, solvent and DI water mixed in defined volume ratios. The concentrations explored ranged from 1:1:1 to 1:4:1 of HF–solvent–DI water. The relevant physicochemical properties of the solvents explored in this work, including ethanol, n-butanol, EG and DMF, are summarized in Table 1. Etching was carried out galvanostatically using a NI PXI-4130 DC power supply (National Instruments, Austin, TX, USA)at a constant current density of 50 mA/cm^2^ for 30 min and at room temperature. After etching, samples were rinsed with IPA, followed by DI water for 3–5 min to remove residual reactants. PL spectra were obtained by irradiating samples with a 365 nm, 100 mW LED light source. Emitted light passed through a filter to remove excitation wavelengths before collection via a USB4000 spectrometer (Ocean Optics, Delray Beach, FL, USA). Morphology was examined by scanning electron microscopy (SEM) using a JEOL JSM-6700F (Japan Electron Optics Laboratory Co., Ltd., Tokyo, Japan) field emission system operated at 5 kV. Both surface and cross-sectional imaging was performed.

## 3. Results and Discussion

### 3.1. Ethanol-Modulated PL and Morphology in Electrochemically-Etched PSi

**Figure 1:**
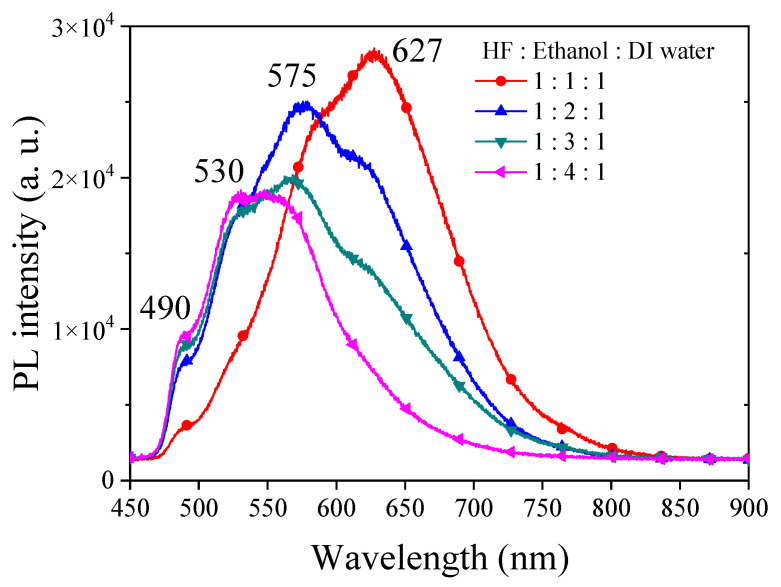
PL spectra of PSi fabricated under various ethanol concentration ratios in an HF-based etching solution.

The visible light PL in PSi is a complex phenomenon with the involvement of multiple factors. Typically, the quantum-confinement effect in PSi is employed to elucidate the origin of its luminescence [24]. When silicon wafers are etched to form PSi structures, the size effect constrains the movement of electrons and holes, leading to alterations in the band structure [25]. This change may result in PL emissions within the visible light range. However, varying degrees of quantum-confinement effects can lead to distinct visible light PL ranges in PSi, with emission wavelengths influenced by the size and morphology of the porous structures. Smaller porous structures are often associated with shorter wavelengths [26]. Defects and surface states on the PSi surface are crucial factors influencing PL. These defects and surface states serve as capture and emission centers for electrons and holes, facilitating PL emission [33]. Additionally, surface defects and states may participate in the radiative recombination process of electrons and holes, giving rise to PL [34]. The characteristics of these surface defects may correspond to specific wavelengths. We first investigated the PL characteristics of PSi under varying ethanol concentrations in a typical HF–ethanol–DI water in volume ratio etching solutions, which yielded insightful results, providing a comprehensive understanding of the interplay between etching conditions and resulting PL properties. Figure 1 shows the experimental results of PL spectra observed from PSi fabricated by adding different concentrations of ethanol. Under HF–ethanol–DI water = 1:1:1 conditions, the PL spectrum exhibited dual peaks at 627 nm (major) and 580 nm (minor). Transitioning to HF–ethanol–DI water = 1:2:1, a substantial blue shift observed from PSi in the major peak to 575 nm was observed, accompanied by a shift in the minor peak to approximately 640 nm. Further elevating the ethanol concentration to HF–ethanol–DI water = 1:3:1 resulted in a continuous blue shift of the central wavelength, coupled with the gradual disappearance of the minor PL peak and a reduction in the full width at half maximum (FWHM) of PL observed from PSi. The observed blue shift with increasing ethanol concentration may suggest a correlation between etching conditions and structural modifications in PSi [35]. The initial presence of multiple peaks at lower ethanol concentrations may be attributed to variations in size and quantum-confinement effects [26]. As the ethanol concentration increases, a more uniform and refined PSi structure may emerge, contributing to the observed blue shift. The reduction and eventual disappearance of the minor peak, as shown in the PSi etched in the etching solution of HF–ethanol–DI water = 1:4:1, suggest alterations in the surface states or defect-related emissions as the ethanol concentration rises [28]. The narrowing of the fluorescence half-width may possibly indicate an improved homogeneity in the PSi structure. The observed multiple emissions in the PL spectra, particularly at approximately 627 nm, 575 nm, 530 nm and 490 nm wavelengths across all samples with varying ethanol concentrations, suggest the presence of different luminescent centers or emission mechanisms within the PSi structure [26]. The 627 nm peak corresponds to the red-shifted emission typically associated with defects or surface states within the PS matrix [27]. These defects can arise from dangling bonds, oxide-related states, or other structural imperfections in the silicon lattice. The red-shifted nature of this emission suggests a lower energy transition, possibly due to the localization of charge carriers near the PSi surface or interfaces [28]. The emission at the 575 nm and 530 nm wavelengths may be attributed to quantum-confinement effects [3] within the PSi nanostructure. As the size of the Si nanocrystallites decreases, quantum confinement leads to an increase in the bandgap energy, resulting in blue-shifted emissions compared to bulk silicon. However, the observed emission wavelength may also be influenced by surface states or defect-related emissions, contributing to the overall PL spectrum. The emission at the 475 nm wavelength may arise from more pronounced quantum-confinement effects or from transitions involving deeper energy levels within the PSi bandgap. Alternatively, this peak could be related to specific chemical species or impurities present in the PS matrix, leading to localized luminescent centers with distinct emission characteristics [2].

The observed blue-shifting of the PL peak with increasing concentrations of ethanol in the etching process of PSi carries significant implications and potential applications. The blue-shifting of the PL peak allows for precise control over the emission wavelength of PSi. By adjusting the concentration of ethanol in the etching solution, researchers can tailor the PL peak to specific wavelengths within the visible spectrum. This tunability is valuable for various optoelectronic applications where specific emission wavelengths are desired, such as LEDs, optical sensors and photonic devices.

**Figure 2:**
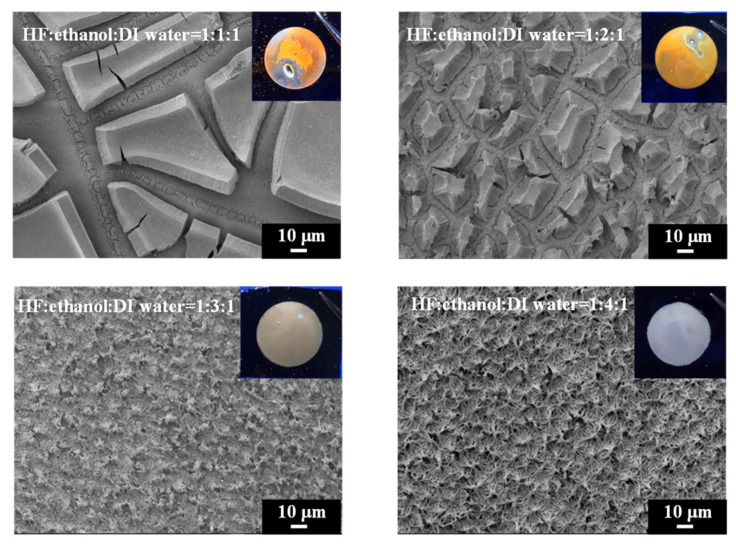
SEM images and corresponding UV-illuminated photographs showing the surface morphology evolution of PSi with increasing ethanol concentrations in the HF-based etching solution.

The SEM images (Figure 2) depict the surface morphology of PSi observed by scanning electron microscopy (SEM) under different etching conditions, with HF–ethanol–water ratios of 1:1:1, 1:2:1, 1:3:1 and 1:4:1. These images provide critical insights into the structural evolution of PSi in response to varying ethanol concentrations in the etching solution. Under HF–ethanol–DI water = 1:1:1 conditions, the PSi surface exhibits a distinctive dual-layer structure. The upper layer comprises micrometer-sized pores, while the lower layer exhibits sub-micrometer to nanometer-sized pores. This phenomenon, previously reported in the literature [33], is attributed to the excessive capillary stress induced by the etching solution on the single-crystal silicon surface, leading to the formation of fractures in the PSi structure [36]. Increasing the etching current could reduce this fracture formation; however, in our study, we demonstrate that increasing the ethanol concentration effectively reduces the surface tension and can help the generation of PSi with a homogeneous surface morphology. Figure 2 also illustrates the SEM image of PSi under HF–ethanol–DI water = 1:2:1 conditions. The micrometer-sized structures in the upper layer noticeably decrease, highlighting the efficacy of the higher ethanol concentration in reducing the surface tension and mitigating fracture formation. This observation aligns with prior research suggesting that the addition of ethanol serves as an alternative strategy to reduce the surface tension during the electrochemical etching of PSi. Continuing the trend, the surface morphology of PSi etched with a HF-ethanol-DI water solution in a ratio of 1:3:1 showed a diminishing trend in the size of the upper-layer micrometer structures, indicating a continuous reduction in capillary stress-induced fractures. Furthermore, a transition towards a singular nanoscale structure is observed in Figure 2, corresponding to HF–ethanol–DI water = 1:4:1 conditions. The complete disappearance of the upper-layer micrometer structures signifies the successful transformation of the PSi surface into a uniform nanoscale morphology. Figure 2 (inset) presents photographic illustrations depicting the etched PSi samples under UV light exposure. The images vividly showcase the impact of the ethanol concentration in HF–ethanol–DI water etching solutions on the visible defects (pores) on the PSi surface. Under conditions of HF–ethanol–DI water = 1:1:1 and 1:2:1, utilizing a lower concentration of ethanol, noticeable visible defects (pores) are observed on the PSi surface, highlighting the influence of the ethanol concentration on the surface quality. The emergence of these visible defects underscores the challenges associated with capillary stress-induced fractures when using lower ethanol concentrations. Contrastingly, when the ethanol concentration is increased in the etching solution (HF–ethanol–DI water = 1:3:1 and 1:4:1), the visible defects on the PSi surface disappear. The enhanced ethanol concentration contributes to the mitigation of capillary stress-induced fractures, resulting in a more pristine and defect-free PSi surface.

**Figure 3:**
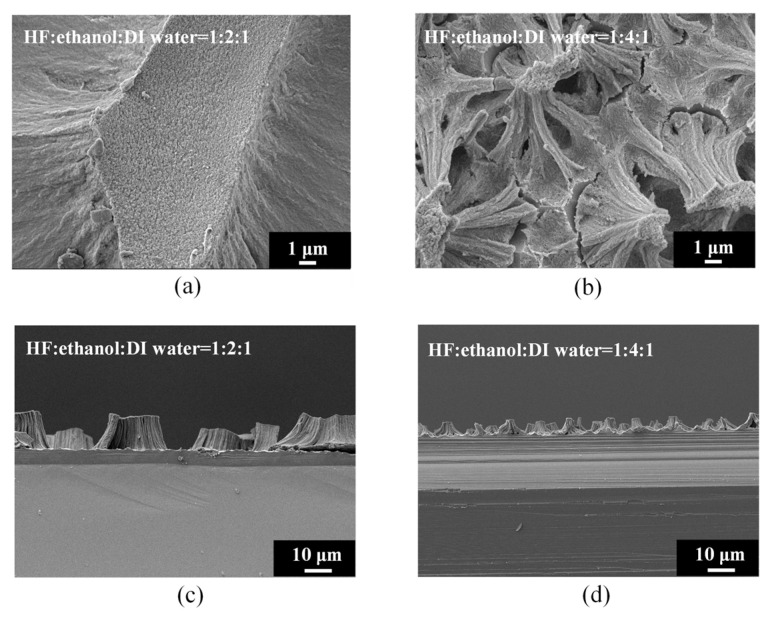
High-magnification SEM images of PSi surfaces (**a**,**b**) and cross sections (**c**,**d**) under different ethanol concentrations in the HF–ethanol–DI water etching solution.

Figure 3 shows the surface and cross-sectional morphology of electrochemically etched PSi. The high-magnification SEM images (Figure 3a,b) provide detailed insights into the surface topography of PSi under distinct etching conditions, specifically HF–ethanol–DI water volume ratios of 1:2:1 and 1:4:1, respectively. Additionally, the corresponding SEM cross-sectional images (Figure 3c,d) offer a comprehensive view of the PSi morphology evolution, emphasizing changes in surface protrusions and etching depth. Under HF–ethanol–DI water = 1:2:1 conditions (Figure 3a), the surface topography of the PSi reveals a significant reduction in the size of the upper-layer protrusions, diminishing from approximately 30 μm to 2 μm. This observation underscores the efficacy of increased ethanol concentrations in refining and minimizing the surface features. Moreover, the SEM cross-sectional image in Figure 3c highlights a noteworthy reduction in the etching height of the PSi, decreasing from 15 μm to 5 μm. This reduction in etching height correlates with the enhanced control over capillary stress-induced fractures, leading to a more uniform and refined PSi structure. By contrast, under HF–ethanol–DI water = 1:4:1 conditions (Figure 3b), the PSi surface demonstrates a homogeneous, non-layered structure, indicating the successful suppression of capillary stress-induced fractures. The corresponding SEM cross-sectional image in Figure 3d further corroborates this observation, revealing a reduced etching height of 5 μm. The elimination of layering and the establishment of a uniform structure highlight the superior control achieved through higher ethanol concentrations in the etching solution.

The observed morphological transitions induced by different concentration of ethanol in the etching process of PSi play a crucial role in determining the structural characteristics and, consequently, the performance of devices based on PSi. These transitions can have a significant impact on device performance, and specific structures may be more desirable for certain applications. The morphology of PSi directly influences its optical and electronic properties, such as light emission, absorption and charge carrier transport. For optoelectronic devices like LEDs and photodetectors, structures with uniform pore distribution and optimized surface roughness are desirable to enhance the light extraction efficiency and reduce non-radiative recombination losses. In biosensing applications, the surface morphology of PSi affects its biomolecular adsorption capacity, surface area-to-volume ratio, and mass transport kinetics. Well-defined pore structures with a high surface area and biocompatible surface chemistry are preferred for biosensors to achieve the sensitive and selective detection of biomolecules.

### 3.2. n-Butanol-Modulated PL and Morphology in Electrochemically-Etched PSi

**Figure 4:**
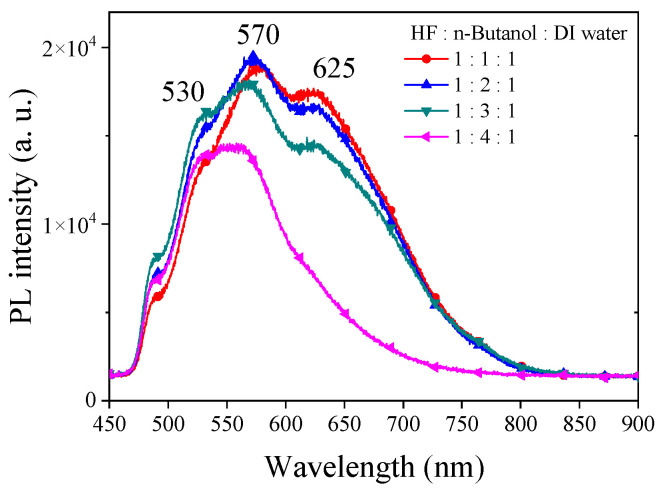
PL spectra of PSi etched using HF-based etching solution using n-butanol solutions at various concentrations.

In our exploration of utilizing n-butanol as a solvent in the etching process of PSi, we maintained all etching conditions identical to those with ethanol. Figure 4 illustrates the PL spectra obtained from PSi etched with different concentrations of n-butanol. Under HF–n-butanol–DI water = 1:1:1 conditions, the PL spectrum of the resulting PSi exhibits a center wavelength of 570 nm. Comparatively, when compared to the same concentration of ethanol, a notable blue shift of approximately 57 nm is observed. Additionally, a secondary emission peak is evident at 625 nm. This observation suggests that the substitution of n-butanol induces a significant impact on the emission characteristics of PSi, with changes in both the main and secondary emission peaks. As the concentration of n-butanol increases, the PL main emission peak remains relatively consistent. Under HF–n-butanol–DI water = 1:4:1 conditions, a slight blue shift of approximately 10 nm is observed in the main emission peak, accompanied by a reduction in the intensity of the secondary emission peak. This trend suggests that higher concentrations of n-butanol do not contribute significantly to altering the main emission characteristics of PSi but may influence the intensity and position of secondary emission peaks.

**Figure 5:**
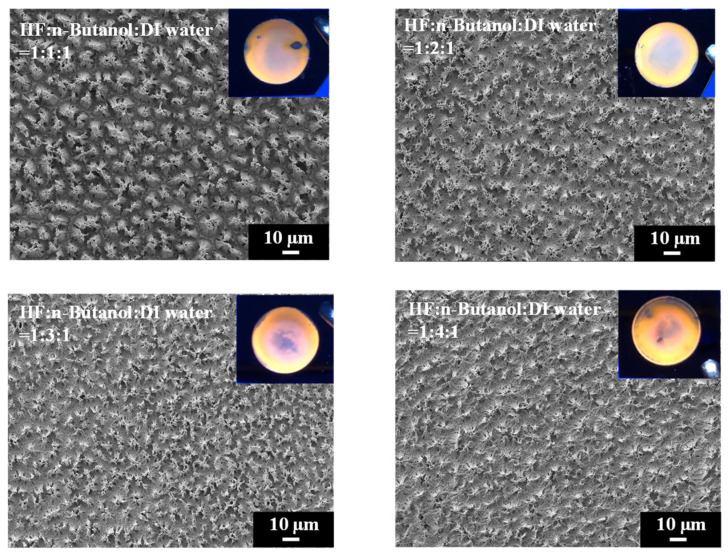
Surface morphology observed from SEM and UV-illuminated photographs of PSi samples etched using different n-butanol solutions.

To gain insights into the structural modifications induced by varying concentrations of n-butanol in the etching process of PSi, SEM analysis was conducted, and the experimental results are shown in Figure 5. Intriguingly, in contrast to the distinctive upper and lower two-layer structure observed when using HF–ethanol–DI water = 1:1:1, the PSi surface etched with HF–n-butanol–water = 1:1:1 exhibits needle-like structures [35,37]. Moreover, as the concentration of n-butanol increases, the diameter of the needle structures decreases. The accompanying photographs under UV exposure (Figure 5, inset) reveal a substantial reduction and disappearance of visible defects on the PSi surface compared to ethanol-etched samples, underscoring the potential advantages of n-butanol as a solvent.

**Figure 6:**
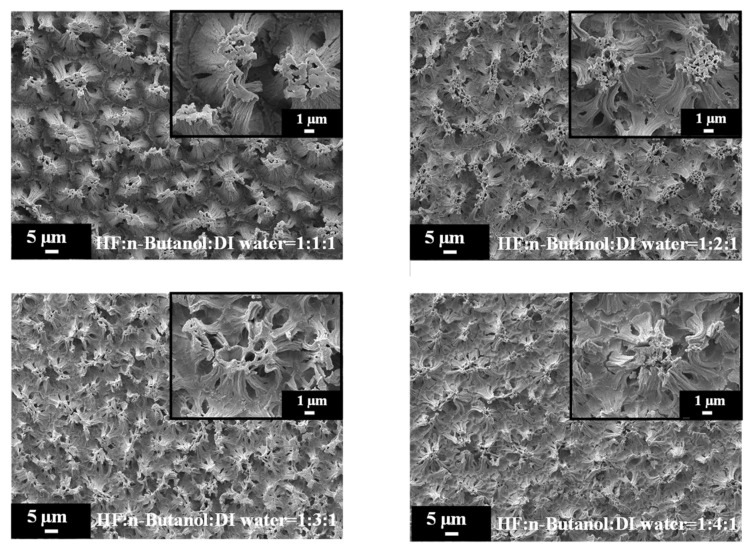
High-magnification SEM images of PSi surface etched with increasing n-butanol concentrations.

**Figure 7:**
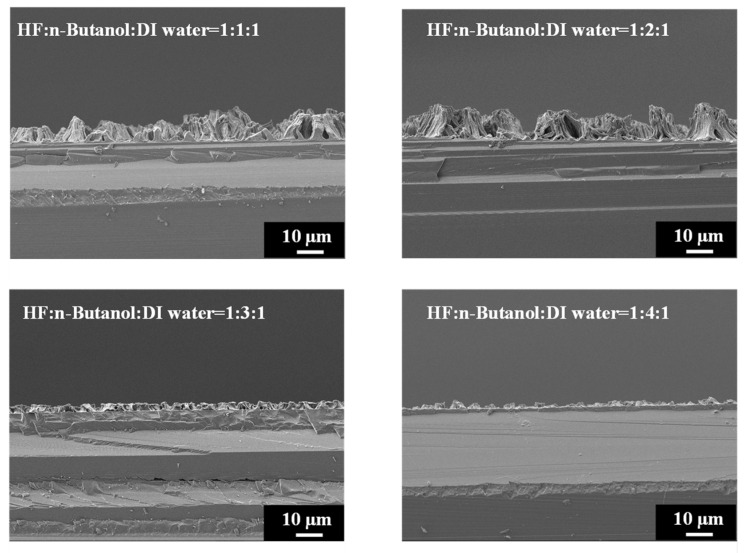
Cross-sectional SEM images exhibiting island-shaped features of PSi fabricated using HF–n-butanol–DI water etching solitons of different n-butanol concentrations.

Figure 6 provides high-magnification SEM images of PSi etched with different concentrations of n-butanol, highlighting the pronounced variations in surface morphology with increasing n-butanol concentrations. The observed changes in the surface morphology are indicative of the intricate interplay between the n-butanol concentration and the resulting structural features of the PSi. Further insight is gained from the SEM cross-sectional images presented in Figure 7. Under HF–n-butanol–water = 1:1:1 and 1:2:1 conditions, the thickness of the porous structure is approximately 10 µm. However, with HF–n-butanol–DI water = 1:3:1, the thickness significantly decreases to 5 µm. Continuing the trend, when using HF–n-butanol–DI water = 1:4:1, the thickness further decreases to 3 µm. This behavior shows similar trends to those observed with ethanol as the solvent.

In our study, a comparative analysis of ethanol and n-butanol as solvents for the etching of PSi reveals similarities in the dielectric constants and dipole moments (Table 1). However, notable distinctions arise regarding the boiling point, vapor pressure and viscosity, with n-butanol exhibiting higher viscosity and lower volatility compared to ethanol. The higher viscosity of n-butanol suggests its potential efficacy in reducing the surface tension during the etching process. This property could facilitate more uniform etching, as increased viscosity tends to mitigate capillary stresses that may otherwise lead to irregularities on the PSi surface. Enhanced uniformity in the etching process is crucial for achieving consistent and reproducible PSi structures. Moreover, the lower volatility of n-butanol compared to ethanol implies a slower rate of evaporation. This reduced evaporation rate may contribute to a more stable etching process, allowing for better control over the formation of PSi structures. Additionally, the diminished volatility could play a role in lowering the surface tension, further supporting the hypothesis of improved etching uniformity. The combination of these solvent properties suggests that n-butanol has the potential to serve as an effective solvent for PSi etching, offering advantages in terms of reduced surface tension and improved process stability. The observed variations in PSi morphology, as discussed in the previous section, align with these solvent-specific characteristics.

### 3.3. EG-Modulated PL and Morphology in Electrochemically-Etched PSi

**Figure 8:**
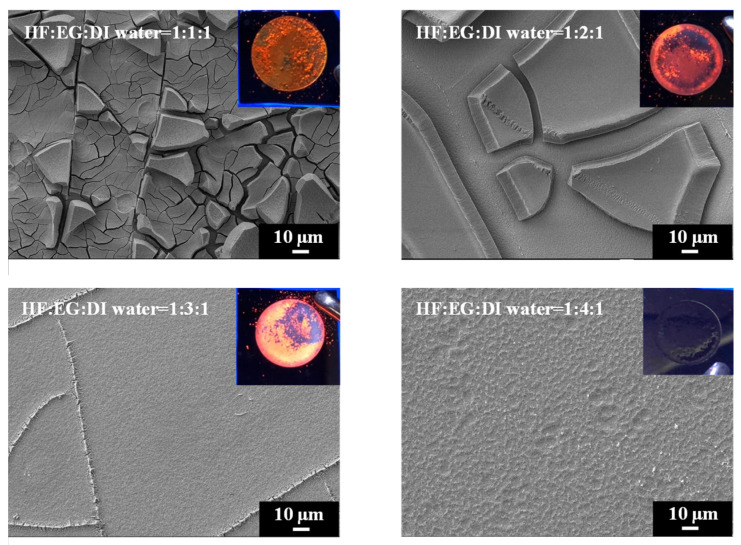
SEM images and corresponding to UV-illuminated photographs exhibiting surface features of PSi fabricated using EG solutions at different concentrations.

**Figure 9:**
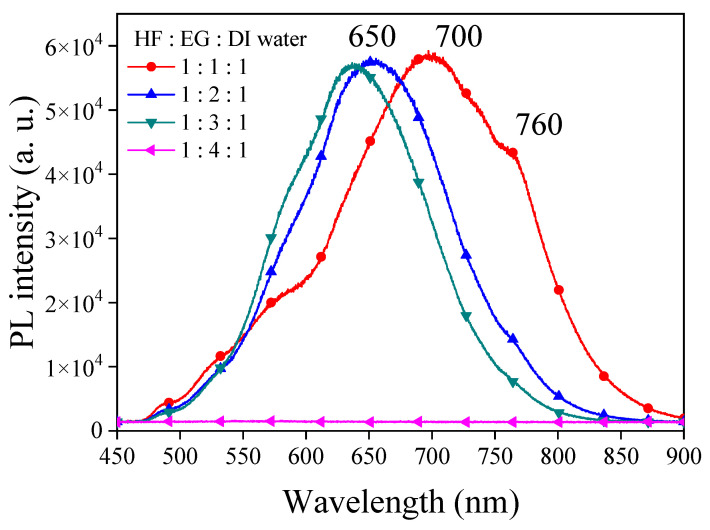
PL spectra of PSi derived from electrochemical etching with EG at varying ratios of HF–EG–DI water etching solution.

We further explored the use of ethylene glycol (EG) as a solvent with exceptionally high viscosity to replace ethanol and n-butanol for reducing the surface tension during PSi etching. The comparison in Table 1 reveals that EG possesses a high dielectric constant, and its dipole moment exceeds those of ethanol and n-butanol. SEM images of PSi surfaces etched with different concentrations of EG are shown in Figure 8. A striking observation arises from the UV-illuminated images of PSi samples, highlighting distinct PL behaviors under different EG concentration ratios. When subjected to the highest EG concentration (HF–EG–DI water = 1:4:1), the PSi sample exhibited no PL (nsert). Conversely, at lower EG concentrations (HF–EG–DI water = 1:1:1), red PL was observed under UV exposure (nsert). However, this condition resulted in visible surface irregularities, manifesting as protrusions. As the EG concentration increased (HF–EG–water = 1:2:1), the PSi sample continued to exhibit red PL under UV illumination, but with a noticeable increase in surface defects (black regions). The black areas correspond to regions where the porous structure detached from the PSi surface, which is similar to over-etching conditions [31]. Further elevating the EG concentration (HF–EG–DI water = 1:3:1) shifted the PL color towards orange, as evidenced in UV-illuminated images (insert). The PL spectra in Figure 9 further support the trend of blue-shifting PL with increasing EG concentrations. At the highest EG concentration (HF–EG–DI water = 1:4:1), the PSi sample exhibited no PL under UV illumination.

**Figure 10:**
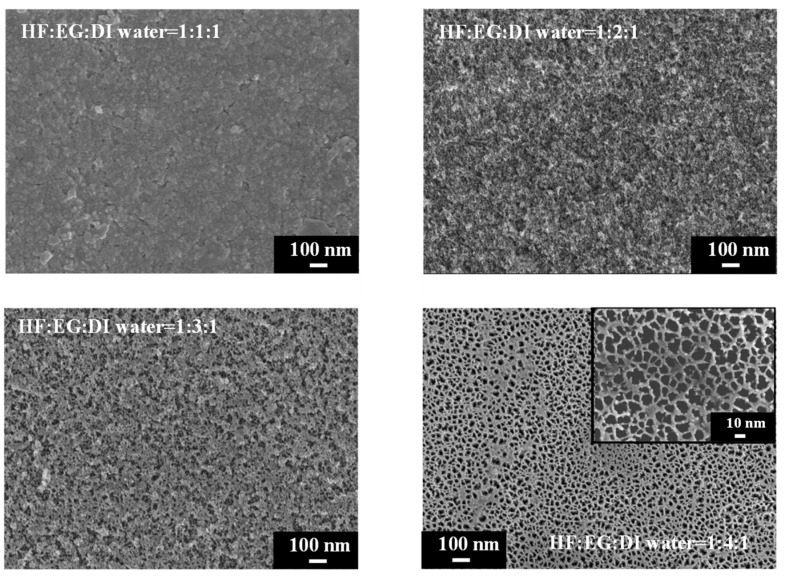
High-magnification SEM micrographs showing pores in the lower porous layer of PSi samples etched using different concentration of EG solutions.

In the examination of the PSi surface morphology, we explored the substitution of ethanol with EG as the solvent during the etching process, aiming for improved control over the PSi structure. The SEM analysis in Figure 8 reveals observations concerning the surface features of PSi samples etched with varying EG concentrations. SEM images, particularly demonstrate that the utilization of EG (HF–EG–DI water = 1:1:1 and HF–EG–DI water = 1:2:1) as a replacement for ethanol results in the formation of a distinct two-layered porous structure on the PSi surface. This bimodal porous structure consists of an upper layer with micron-sized pores and a lower layer with submicron to nanoscale pores. The manifestation of such a morphology is noteworthy, especially when compared to the PSi structures obtained using ethanol as the solvent. The progressive increase in EG concentration during PSi etching (HF–EG–DI water =1:3:1 and 1:4:1) reveals significant alterations in the PSi morphology, as shown in the SEM images. An analysis indicates the disappearance of the upper micron-sized structures, leaving only the lower nanoscale porous features as the EG concentration continues to rise. Further examination at a higher magnification, as illustrated in Figure 10, provides insights into the size of the pores within the lower layer structure. Interestingly, at lower EG concentrations, no pores are discernible in the lower layer structure, implying that the PL of the PSi primarily originates from the upper-layer porous structure. With the gradual increase in EG concentration, the upper layer structure diminishes in size, resulting in a blue shift in the PL center wavelength. Notably, as the EG concentration reaches HF–EG–water = 1:4:1, the upper layer structure completely disappears. Under this etching condition, EG etching then transitions to primarily affect the lower layer, creating nano-sized pores (Figure 10, HF–EG–water = 1:4:1 inset). The emergence of approximately 20 nm pores, while significant for the overall morphology, does not significantly contribute to the PL from the PSi. This nuanced interplay between the EG concentration and PSi morphology highlights the intricate relationship between etching conditions and the resulting optical properties of PSi.

### 3.4. DMF-Modulated PL and Morphology in Electrochemically-Etched PSi

**Figure 11:**
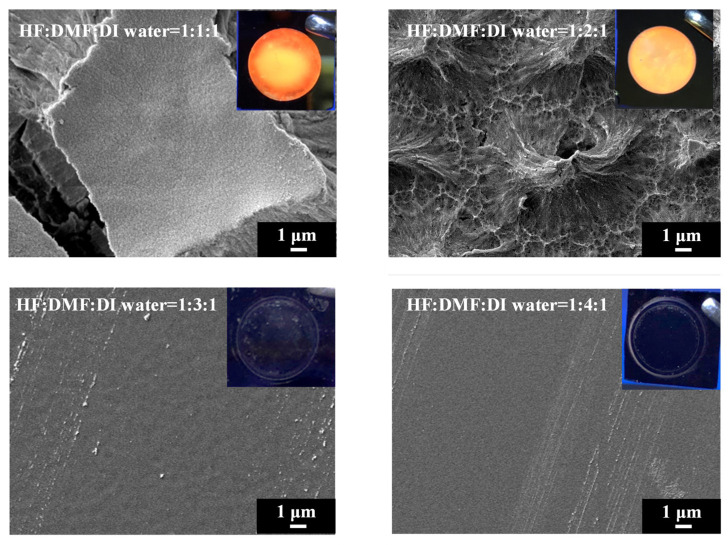
SEM images and corresponding UV-illuminated photographs of PSi etched electrochemically with the mixture of HF–DMF–DI water at different DMF concentrations.

**Figure 12:**
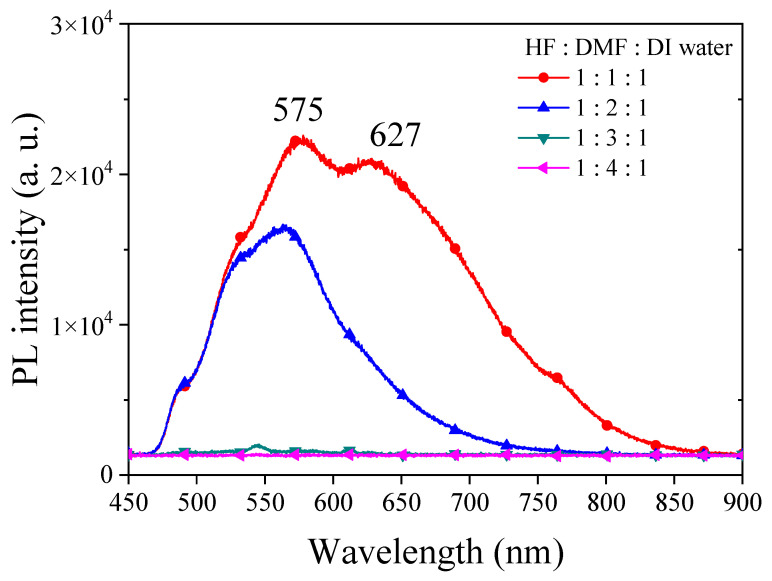
PL spectra of PSi fabricated by etching silicon using an HF–DMF–DI water etching solution at various volume ratios of DMF.

In order to investigate the influence of solvent properties such as the dielectric constant and dipole moment on the electrochemical etching of PSi with HF, we employed DMF, a solvent with a dielectric constant comparable to EG but with lower viscosity. Different concentrations of DMF were utilized in HF-based etching processes, and the resulting PSi was characterized through UV irradiation, surface SEM imaging and corresponding PL spectra, as illustrated in Figure 11 and Figure 12. Under HF–DMF–DI water = 1:1:1 conditions, the SEM images reveal a bi-layered porous structure reminiscent of ethanol-added etching, with no visible defects in the UV irradiation photograph (inset). Upon increasing the DMF concentration to HF–DMF–DI water = 1:2:1, a notable transition occurred, leading to the disappearance of the upper layer structure, forming needle-like features similar to those observed with high concentrations of ethanol and n-butanol. In this condition, the PSi exhibited no visible defects under UV light (inset). Further increasing the DMF concentration to HF–DMF–water = 1:3:1 and HF–DMF–water = 1:4:1 resulted in the disappearance of both the PL emission and the observable porous structures in the SEM images. The use of DMF as a diluent for HF in silicon substrate etching to form PSi provides interesting insights into the resulting surface morphology. In comparison with ethanol, when DMF is employed, it becomes evident that at suitable concentrations, notably HF–DMF–water = 1:2:1, a uniform and well-defined needle-like porous structure can be easily achieved. This suggests that, within specific concentration ranges, DMF facilitates the creation of a homogeneously etched PSi surface. However, a crucial observation emerges as the DMF concentration continues to increase. Unlike the trends observed with ethanol and n-butanol, a continuous increase in DMF concentration leads to an undesired outcome resembling the effects seen with EG. The over-etching results in the detachment of the porous structure from the substrate, indicating a critical concentration control requirement when utilizing highly polar solvents. This behavior, which is distinct from ethanol and n-butanol trends, underscores the nuanced impact of the solvent polarity on the etching process. While ethanol and n-butanol demonstrate a controlled etching response with rising concentrations, the impact of DMF is characterized by increased intricacy. The polarity and molecular interactions of DMF necessitate precise concentration tuning to attain the desired uniformity in PSi morphology.

Based on the observation of DMF-etched PSi samples, we can infer that highly polar organic solvents may effectively wet the silicon surface, enhancing the diffusion and action of the etchant and leading to an increased etching rate. This could result in a faster etching process. Due to the higher solvent polarity, it may impact the PL characteristics of PSi. Changes in solvent polarity may alter the PL peak position, intensity and waveform. The polarity and volatility of the solvent may influence the surface tension during the etching process, thereby affecting the surface morphology of the PSi. Solvents with greater polarity could potentially modify the formation mechanism of PSi structures. The solvent properties may impact the selectivity of etching rates on different crystal faces, influencing the final morphology and structure of PSi. More polar organic solvents may enhance the affinity of the liquid to the silicon surface, facilitating more uniform surface coverage and reducing potential uneven etching. However, the excessive participation of polar solvents can lead to overetching, preventing the formation of porous structures on the silicon substrate surface. The findings suggest that the use of highly polar solvents requires careful consideration of concentration parameters in order to achieve controlled and uniform etching. The propensity of DMF to induce excessive etching at higher concentrations indicates the need for a nuanced approach to solvent selection and concentration tuning in the fabrication of PSi structures.

### 3.5. Inference of Solvent Properties on the Etching Process and Resultant Properties

The observed influence of solvents on the etching process and resultant properties of PSi can be attributed to several mechanistic insights, which may vary depending on the specific solvent properties such as polarity, volatility and viscosity. The polarity of solvents can significantly impact the dissolution kinetics and surface interactions during PSi etching. Polar solvents like DMF may enhance the wetting of Si surfaces and facilitate the transport of etchant species, leading to more uniform and controlled etching. Conversely, nonpolar solvents such as n-butanol may exhibit different etching behaviors, potentially resulting in nonuniform pore formation or surface defects due to differences in surface affinity and dissolution rates. The volatility of solvents affects their evaporation rates during the etching process, which can influence the concentration of etchant species at the PSi surface. Solvents with higher volatility may promote faster etching rates but could also lead to nonuniform etching or surface roughening if not properly controlled. Conversely, less-volatile solvents may provide more stable etching conditions, allowing for better control over pore morphology and surface characteristics. The viscosity of solvents plays a crucial role in controlling the fluid dynamics and mass transport during the etching process. Higher-viscosity solvents, such as EG and DMF, may exhibit slower diffusion rates of etchant species, resulting in more gradual and controlled etching compared to lower-viscosity solvents like ethanol or n-butanol. However, excessively high viscosity may hinder the fluid flow and lead to incomplete pore formation or surface irregularities. Solvent properties such as the surface tension and surface passivation ability also influence PSi etching. Solvents with a lower surface tension may better wet the silicon surface, facilitating more uniform pore nucleation and growth. The interplay of these solvent properties determines the etching kinetics, pore morphology, surface structure, and PL behavior of PSi. Mechanistic insights into solvent effects on PSi etching can provide valuable guidance for optimizing etching processes and tailoring PSi properties for various applications.

## 4. Conclusions

Our work presents a systematic investigation on the effects of various organic solvents and their concentrations on the PL and surface morphology of PSi fabricated through electrochemical etching. The results provide several valuable insights. Increasing ethanol and n-butanol concentrations induces a continuous blue shift in the main PL emission peak of PSi, indicating changes in the quantum-confinement effects related to structural refinements. Meanwhile, secondary defect-related peaks diminish. n-butanol only slightly impacts the main PL peak position compared to ethanol; however, it still facilitates uniform etching at higher concentrations. Excessive etching is observed for the highly polar solvents EG and DMF at higher concentrations, disrupting the formation of the porous layer and quenching PL. Complex interplays exist between solvent parameters such as polarity, viscosity and volatility and their influence on the surface wetting capability, diffusivity and etching kinetics. This governs the morphological evolution of PSi. Further exploration of solvent types beyond those studied in this research could provide additional insights into their effects on PSi properties. Exploring solvents with different polarities, volatilities and viscosities may uncover novel etching mechanisms and lead to the development of optimized etching processes. Experimenting with various solvent ratios and compositions could help fine-tune the etching process to achieve specific pore morphologies and surface structures, exploring the potential applications of solvent-modified PSi in various fields, such as optoelectronics, sensing and biomedical devices. Investigating the performance of solvent-treated PSi in specific applications could uncover new functionalities and performance enhancements enabled by solvent engineering. Overall, our work highlights the critical role of solvent selection and concentration tuning in electrochemically engineered PSi to achieve optimized PL properties and surface morphologies. The findings provide directive guidelines for designing tailored solvent-based etching processes for enhanced performance in PSi-based optoelectronic devices.

## Figures and Tables

**Table 1:** Physicochemical properties of solvents employed in electrochemical etching of PSi, including the dielectric constant, dipole moment, viscosity, boiling point and vapor pressure.

	Dielectric Constant	Boiling Point (°C)	Vapor Pressure 20 °C (hPa)	Dipole Moment (D)	Viscosity (10^−3^ Pa·s)	Solubility in Water (g/100g)
n-Butanol	17.8	117.7	6.3	1.7	2.59	7.7
Ethanol	24	78.5	59	1.7	1.08	Miscible
EG	37.7	197	16.1	2.3	16.1	Miscible
DMF	38.3	153	3.5	3.8	0.80	Miscible
Water	80	100	17.5	1.85	0.89	Miscible

## Data Availability

Data are contained within the article.
